# Immediate implant placement in the posterior mandibular region was assisted by dynamic real-time navigation: a retrospective study

**DOI:** 10.1186/s12903-024-03947-x

**Published:** 2024-02-09

**Authors:** Ningbo Geng, Jing Ren, Chi Zhang, Tianren Zhou, Chongjin Feng, Songling Chen

**Affiliations:** Department of Stomatology, The First Affiliated Hospital of Sun Yat-sen University, Guangzhou, 510080 China

**Keywords:** Immediate placement, Computer-assisted surgery, Virtual implant planning, Guided surgery, Retrospective study

## Abstract

**Background:**

Efficient utilization of residual bone volume and the prevention of inferior alveolar nerve injury are critical considerations in immediate implant placement (IIP) within the posterior mandibular region. Addressing these challenges, this study focuses on the clinical efficacy and implant accuracy of dynamic real-time navigation, an emerging technology designed to enhance precision in implantation procedures.

**Methods:**

This study included 84 patients with 130 implants undergoing immediate placement in the posterior mandibular region. Stratified into dynamic navigation, static guide plate, and freehand implant groups, clinical indicators, including initial stability, distance to the inferior alveolar nerve canal, depth of implant placement, and various deviations, were systematically recorded. Statistical analysis, employing 1- or 2-way ANOVA and Student’s t-test, allowed for a comprehensive evaluation of the efficacy of each technique.

**Results:**

All 130 implants were successfully placed with an average torque of 22.53 ± 5.93 N.cm. In the navigation group, the distance to the inferior alveolar nerve and the depth of implant placement were significantly greater compared to the guide plate and freehand groups (*P* < 0.05). Implant deviation was significantly smaller in both the navigation and guide plate groups compared to the freehand group(*P* < 0.05). Additionally, the navigation group exhibited significantly reduced root and angle deviations compared to the guide plate group(*P* < 0.05), highlighting the superior precision of navigation-assisted immediate implant placement.

**Conclusions:**

It is more advantageous to use dynamic navigation rather than a static guide plate and free-hand implant insertion for immediate posterior mandibular implant implantation.

## Background

In comparison to conventional implant placement, immediate implant placement (IIP) offers the advantages of reducing the number of operations, shortening the time required for implant restoration, and preserving both soft and hard tissues to the utmost extent [1, 2]. Simultaneously, when strictly adhering to indications, the success rate of immediate implant placement (IIP) is comparable to that of conventional implantation [3, 4]. At present, immediate implant placement (IIP) was predominantly employed in the anterior region of single-rooted teeth and narrow alveolar sockets in premolar areas post-extraction. However, limited research exists on its application in multi-rooted posterior teeth with larger alveolar sockets. Ensuring primary stability in IIP becomes challenging in molar teeth due to their extensive alveolar fossa and the morphological mismatch with the implant [5]. Moreover, insufficient bone in the posterior mandibular region for immediate implant placement (IIP) poses a potential risk of damaging the inferior alveolar nerve [6].

Conventional free-hand implantation demands advanced surgical skills. Additionally, the limited visibility in the mandibular posterior region affects the surgeon’s judgment of the anatomical conditions, resulting in increased potential for significant implantation deviation and an elevated risk of nerve injury [7].

In recent years, there has been a gradual application of digital guide plates and navigation technology in oral implantation. Thanks to its benefits, including preoperative digital implant design and precise intraoperative control, this approach significantly reduces the technical challenges associated with implantation [8]. While the use of guide plates enhances the accuracy of immediate implantation in mandibular posterior teeth, certain issues persist. Challenges include the impact of water cooling, potentially leading to bone burns. Additionally, the guide plate affects the visual field and complicates implant operations. Furthermore, its application is limited when the patient’s mouth opening is insufficient [9, 10].

Dynamic real-time navigation has emerged as a recent technological advancement in dental implantology. This technology offers real-time feedback on the drilling and implant placement path during the procedure, boasting high accuracy and visualization benefits. Notably, it addresses challenges related to insufficient mouth opening in the posterior mandibular region. Moreover, the dynamic navigation system provides real-time displays of the implantation path and the location of the inferior alveolar nerve canal during the entire implantation process [11, 12].

Hence, this study implemented dynamic real-time navigation to aid in immediate implant placement (IIP) in the posterior mandibular region. The research assessed the accuracy and relevant indicators of IIP in this region, comparing the outcomes with those of the guide plate and free-hand groups. The objective was to evaluate the clinical significance of navigation in the context of IIP in the posterior mandibular region.

## Materials and methods

### Study design and patient cohort

The sample size calculation was performed using PASS v15 software, employing a one-way ANOVA statistical test. The mean deviation of placed implants from the studies of Jorba et al. [13] and Block et al. [14] was utilized to determine the effect size, resulting in a value of 0.42. With a significance level (α) set at 0.05 and a desired power (1–β) of 0.8, a recommended sample size of 35 implants per group was determined. To account for potential dropouts, an additional 25% was added, establishing a total sample size of 130 implants, divided into three groups.

A retrospective enrollment included 84 patients (43 males and 41 females) who underwent immediate implant placement in the mandibular posterior region at the Department of Stomatology between January 2021 and December 2022. The age range of patients was 20 to 78 years, with a mean age of (46.25 ± 13.86) years. A total of 130 tapered implants (Nobel Biocare, Sweden; Straumann, Switzerland) were inserted, with the study divided into three groups: dynamic navigation (28 patients, 40 implants), static guide (26 patients, 44 implants), and freehand implant group (30 patients, 46 implants) as detailed in Table 1. Approval for the study was obtained from the Ethics Committee of the First Affiliated Hospital of Sun Yat-sen University (No. [2022]104).

**Table 1:** The demographic and measurement data for the study (*N* = 130)

Variables	Navigation group	Guide plate group	Free hand group	F-value	P
Number of subjects	28	26	30		
Sex (max/females)	16/12	12/14	16/14		
Age	45.8 ± 16.48	50.32 ± 19.24	48.75 ± 15.63	1.865	0.37
Distance from nerve(mm)	2.68 ± 0.50	1.97 ± 0.17	1.21 ± 0.33	12.51	0.0072**
Implant depth (mm)	12.73 ± 0.93	11.26 ± 0.69	10.24 ± 1.41	5.574	0.0266*
neck deviation(mm)	0.55 ± 0.08	0.73 ± 0.10	1.28 ± 0.12	21.66	0.0018**
root deviation(mm)	0.52 ± 0.13	1.33 ± 0.42	2.22 ± 0.30	23.31	0.0015**
depth deviation(mm)	0.43 ± 0.14	0.49 ± 0.11	0.53 ± 0.09	1.009	0.4191
angle deviation	0.88 ± 0.45	1.77 ± 0.30	3.52 ± 1.03	12.87	0.0068**

*P < 0.05; **P < 0.01

#### Inclusion criteria

Patients meeting the inclusion criteria for this study were those who underwent tooth extraction in the posterior mandibular region due to endodontic and periodontal diseases. Adequate mouth opening, allowing for the accommodation of the navigation registration device and implant guide plate, was a prerequisite. Preoperative CBCT scans were required to show a minimum of 3 mm of available bone for implantation from the root apex to the inferior alveolar nerve canal. Additionally, individuals included in the study had no evidence of acute inflammation in the teeth, and a consistent implant torque of 15 N.cm was successfully achieved in all implants.

#### Exclusion criteria

Patients were excluded from the study if they had severe systemic diseases that would compromise their ability to tolerate implant surgery. The presence of severe local apical and periodontal diseases also constituted exclusion criteria. Furthermore, individuals with a significant limitation of mouth opening, preventing the accommodation of the navigation registration device and implant guide plate, were not included in the study.

### Clinical process

#### Preoperative digital implant planning

**Fig. 1:**
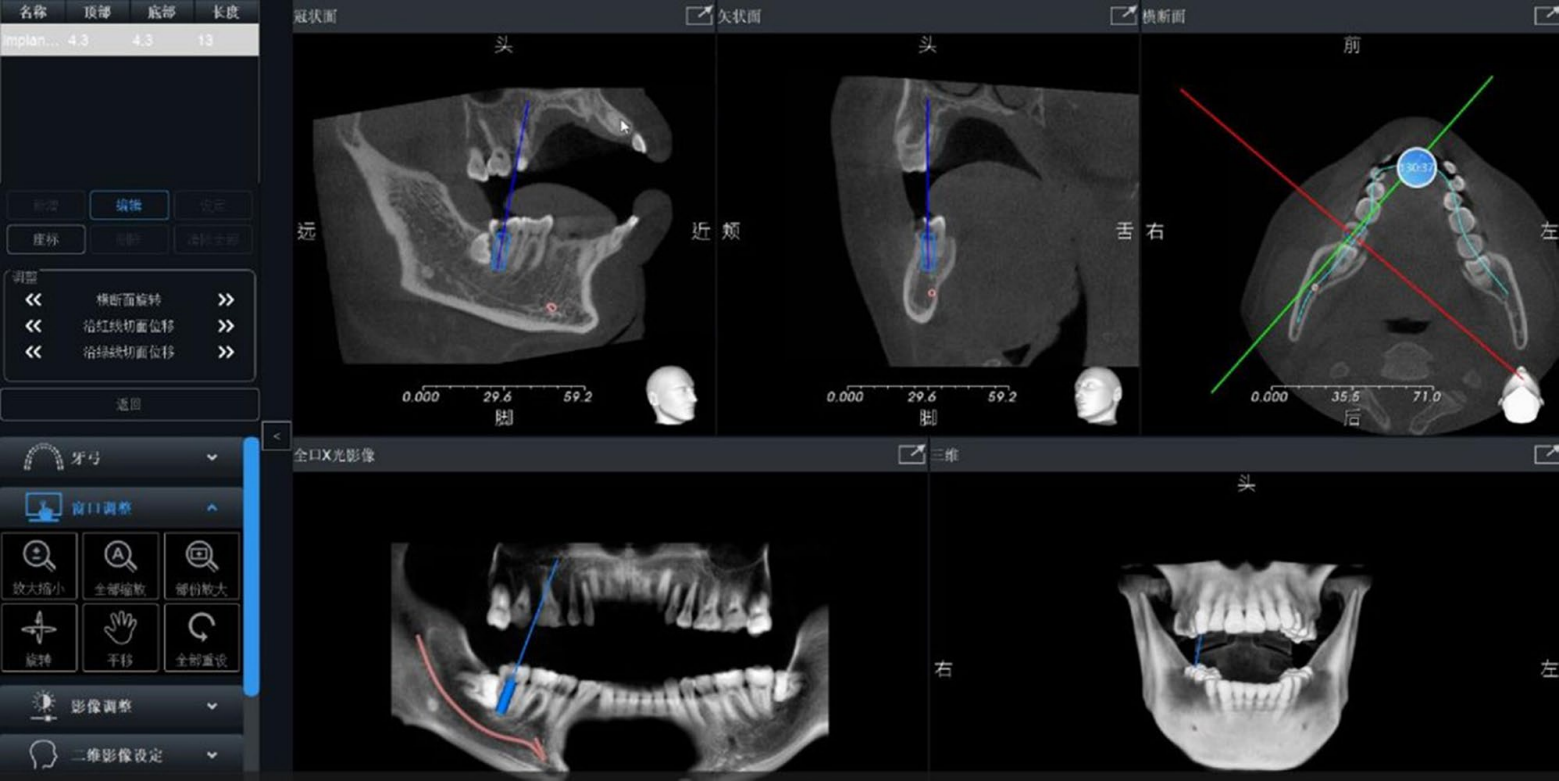
The inferior alveolar nerve canal was drawn and the implant plan was designed virtually

Cone-beam CT (CBCT) scans of the maxillofacial region were conducted using iCAT imaging equipment (Imaging Sciences International, Inc., Hatfield, USA). The acquired CBCT data were imported into implant design software, specifically In-vivo 5 by Anatomage (USA), as illustrated in Fig. 1. Within the software, the jaw model was meticulously reconstructed, and the configuration of the inferior alveolar nerve was delineated. Subsequently, an appropriate implant was selected based on individual requirements, and the three-dimensional position of the implant was meticulously designed. This advanced process facilitated precise planning and optimization of the implantation path.

In the dynamic navigation group, a positioning registration device was crafted prior to the operation, and intraoperative registration was conducted by selecting feature points using the Iris-100 system(Iris-100, EPED Group, Taiwan). This system facilitated accurate and real-time tracking during the surgical procedure, enhancing the precision of implant placement in the posterior mandibular region.

Within the static guided group, the guide plate models underwent the production of implant guide plates using a guide 3D printing device. These guide plates were specifically designed for the group using guide design software (3Shape, DM700, Denmark). Following the design process, both the implant model and guide plate model were saved as STL files. The final step involved utilizing a 3D guide printer to fabricate the implantation guide, ensuring precision and adherence to the planned implantation path during the subsequent surgical procedure.

#### Dynamic real-time navigation assisted implant surgery

Following the completion of disinfection, a minimally invasive extraction of the affected tooth was performed in preparation for implant placement. Special attention was dedicated to preserving the integrity of the bone wall of the extraction socket throughout the procedure. This cautious approach aimed to maintain optimal conditions for subsequent implantation in the posterior mandibular region.

**Fig. 2:**
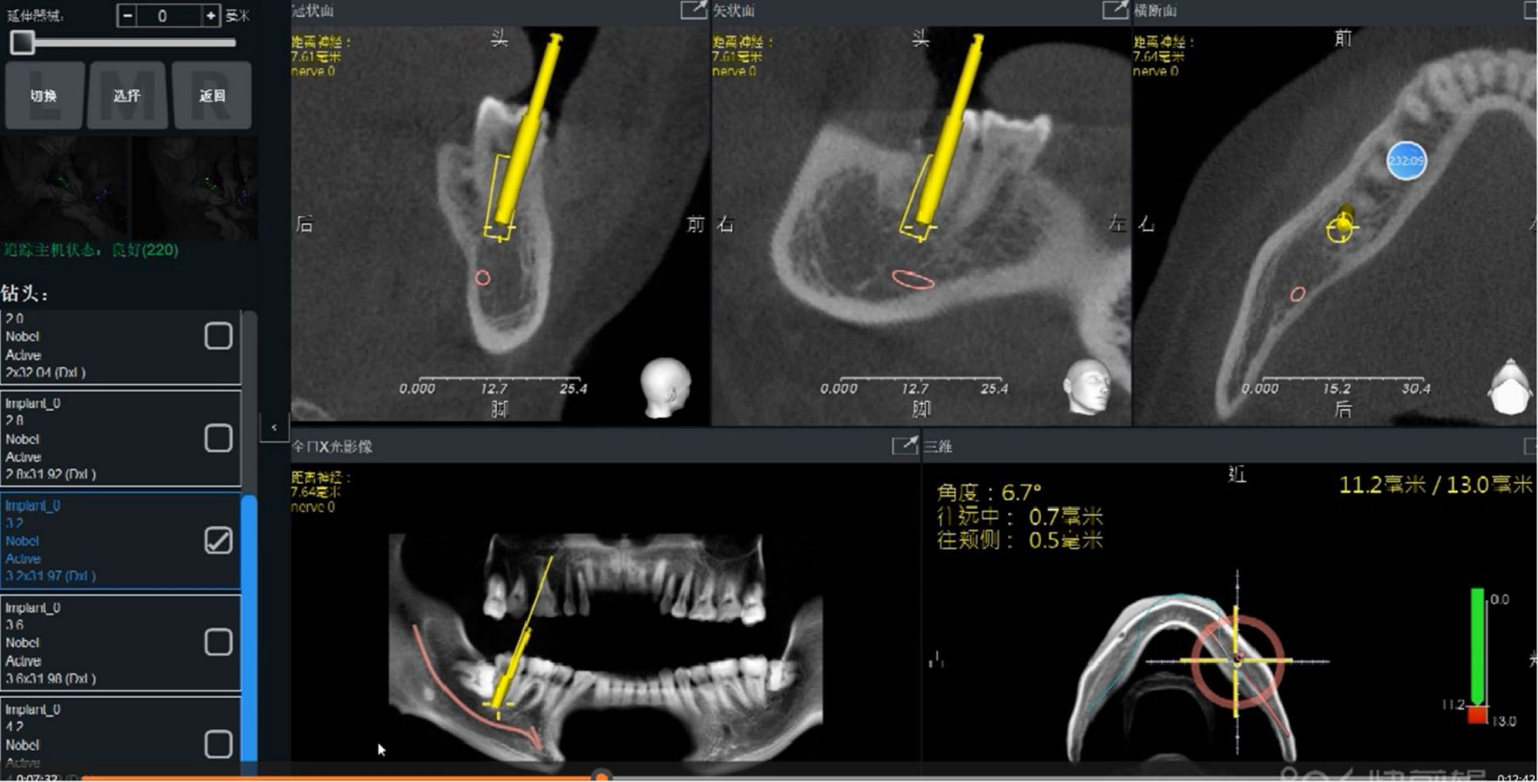
Dynamic navigation guided the immediate implantation of the mandibular posterior teeth

Firstly, the navigator(Iris-100, EPED Group, Taiwan)was positioned and connected to the instrument. Infrared tracking was applied to calibrate the dental handpiece for implantation. An infrared tracking device was used to calibrate the implant handpiece, and a registration device was placed in the mandibular posterior tooth area to confirm the stability of the retainer and register the feature points. After registration, the position relationship between the implant handpiece and the patient’s jaw was displayed on the display screen of the navigator. Under the guidance of dynamic real-time navigation system, dental implant surgery was performed by the same physician (Fig. 2).

**Fig. 3:**
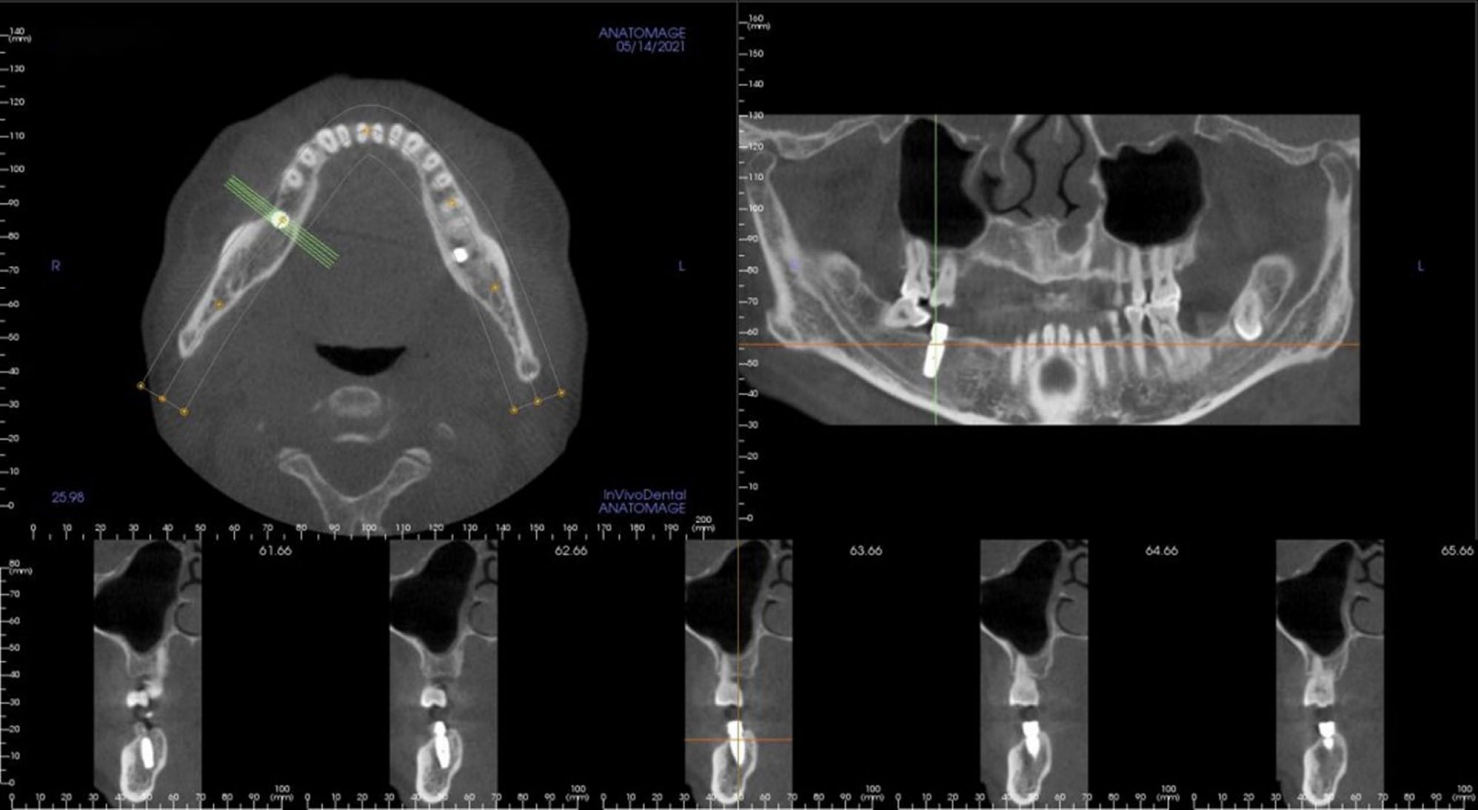
CBCT of dynamic navigation assisted immediate implantation of the mandibular posterior teeth region

After implant preparation, a suitable implant was placed in the implant socket and a healing abutment or a cover screw was installed. Finally, the wound sutured. Finally, under the guidance of dynamic navigation, the implants were accurately placed in the implant socket effectively avoiding the inferior alveolar nerve canal (Fig. 3).

#### Static guide assisted implant surgery

**Fig. 4:**
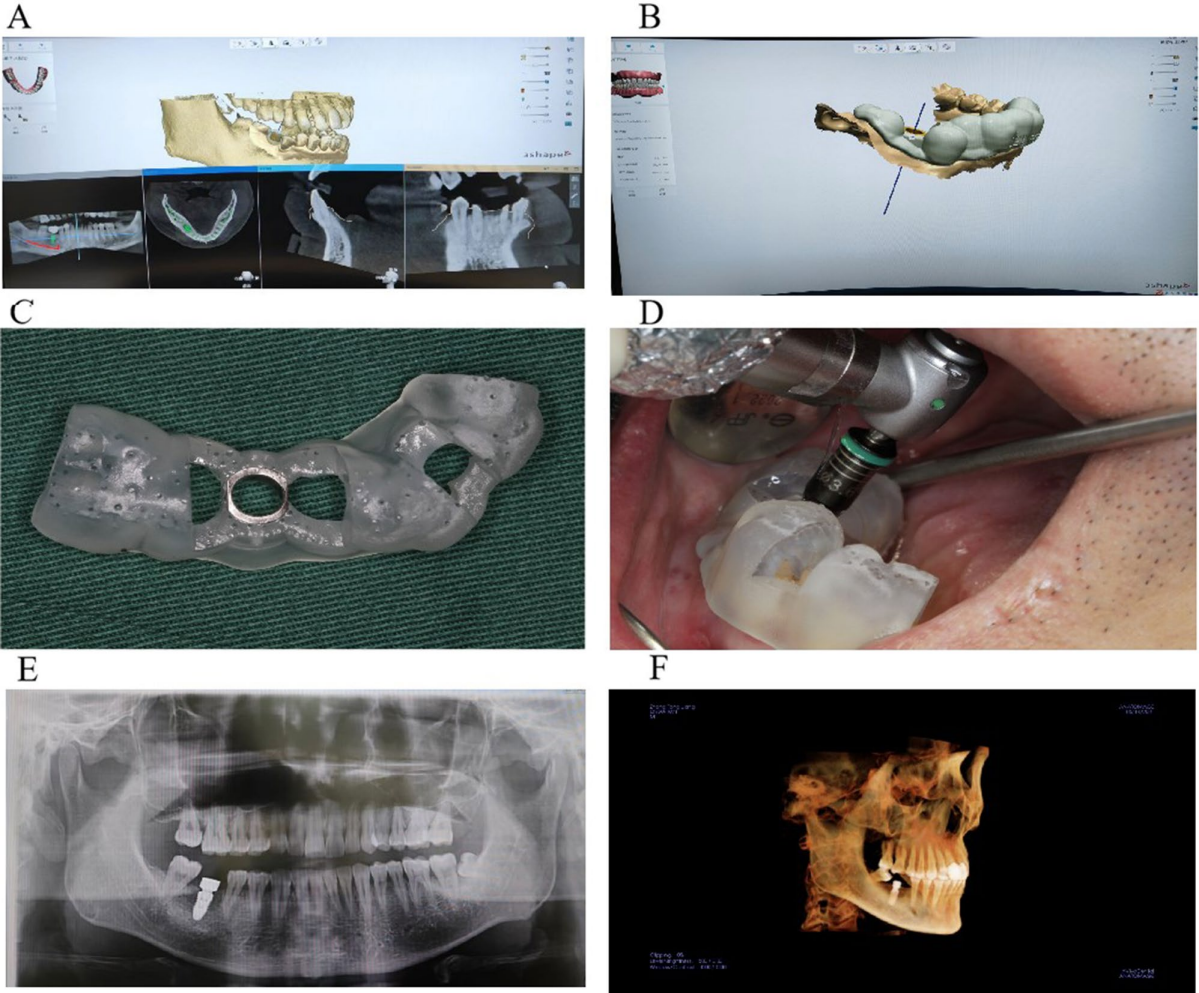
The procedure of mandibular posterior dental implant treatment assisted by static guide plate. (**A**) preoperative implant design; (**B**) Design of implant guides; (**C**) mandibular implant guide plate; (**D**) placement of implant guide plate during operation; (**E**) postoperative panorama; (**F**) Postoperative three-dimensional reconstruction

Following the minimally invasive extraction of affected teeth, the implant guide plate was securely positioned without any tilting. Utilizing the pioneer drill and reaming drill under the precise guidance of the implant guide plate, the implant cavity was meticulously prepared. Subsequently, an appropriately sized implant was placed into the prepared socket, and either a healing abutment or a cover screw was installed, as depicted in Fig. 4. This step-by-step process ensured accurate implant placement and set the foundation for the subsequent stages of the implantation procedure.

#### IIP in the posterior mandibular region of the freehand

**Fig. 5:**
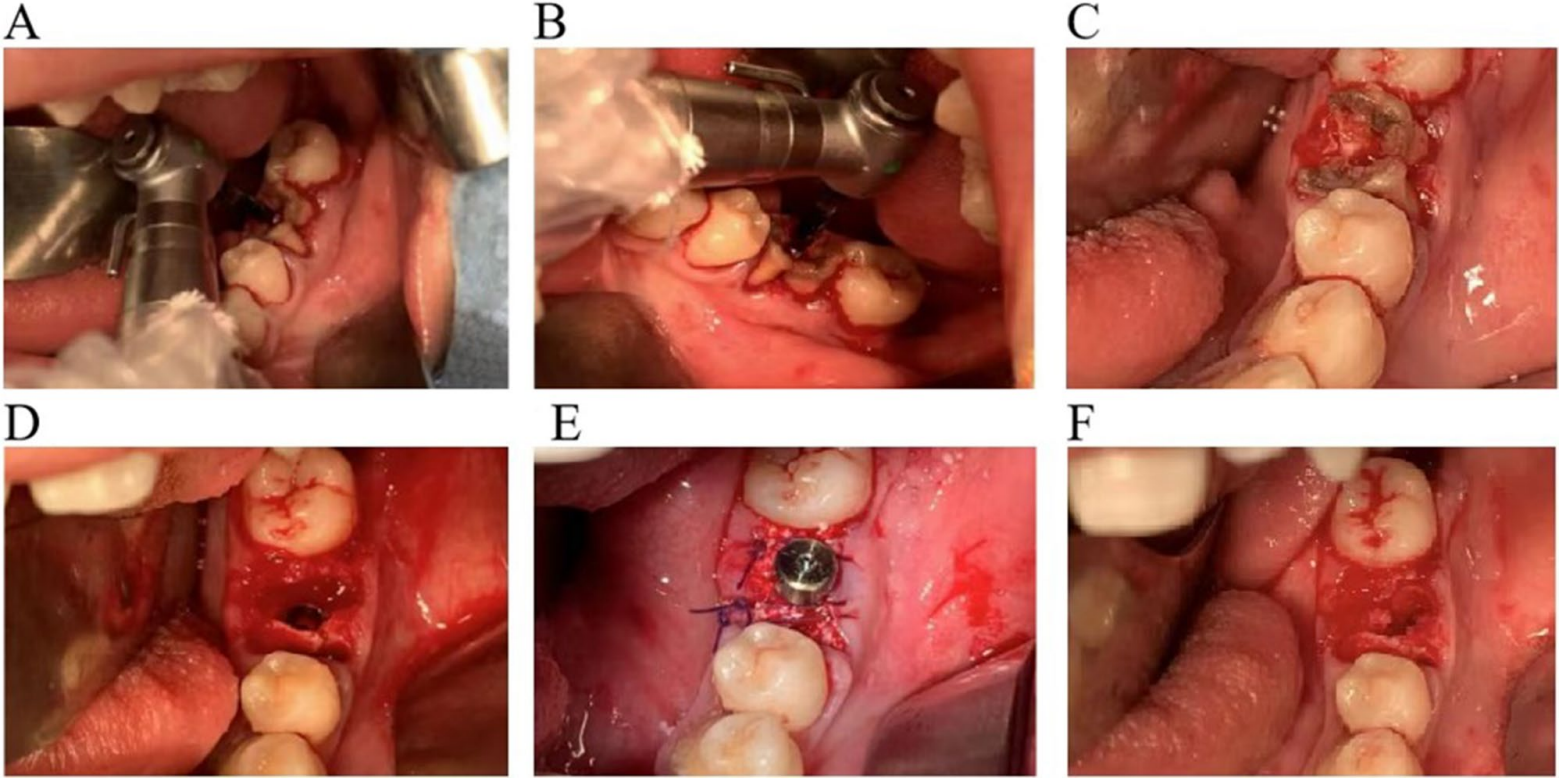
Free-hand immediate implantation process

Following disinfection, the crown of the tooth was removed, and the root was retained. The implant point was identified by assessing the spacing among the roots, and the implant preparation was conducted at the root or root furcation. Upon completion of the preparation, the implant was carefully placed. Subsequently, a healing abutment or cover screw was installed, and the wound was securely sutured, as illustrated in Fig. 5. This meticulous process ensured proper implant placement and facilitated optimal healing conditions.

All surgical procedures were consistently performed by the same operator to maintain uniformity. Following surgery, all patients received routine anti-infective therapy. Custom abutments and all-ceramic crowns were subsequently fabricated and delivered approximately five months after the initial implant placement. This approach ensured standardized care and allowed for optimal healing and integration before the final restoration.

### Observation indicators and measurement standards

**Fig. 6:**
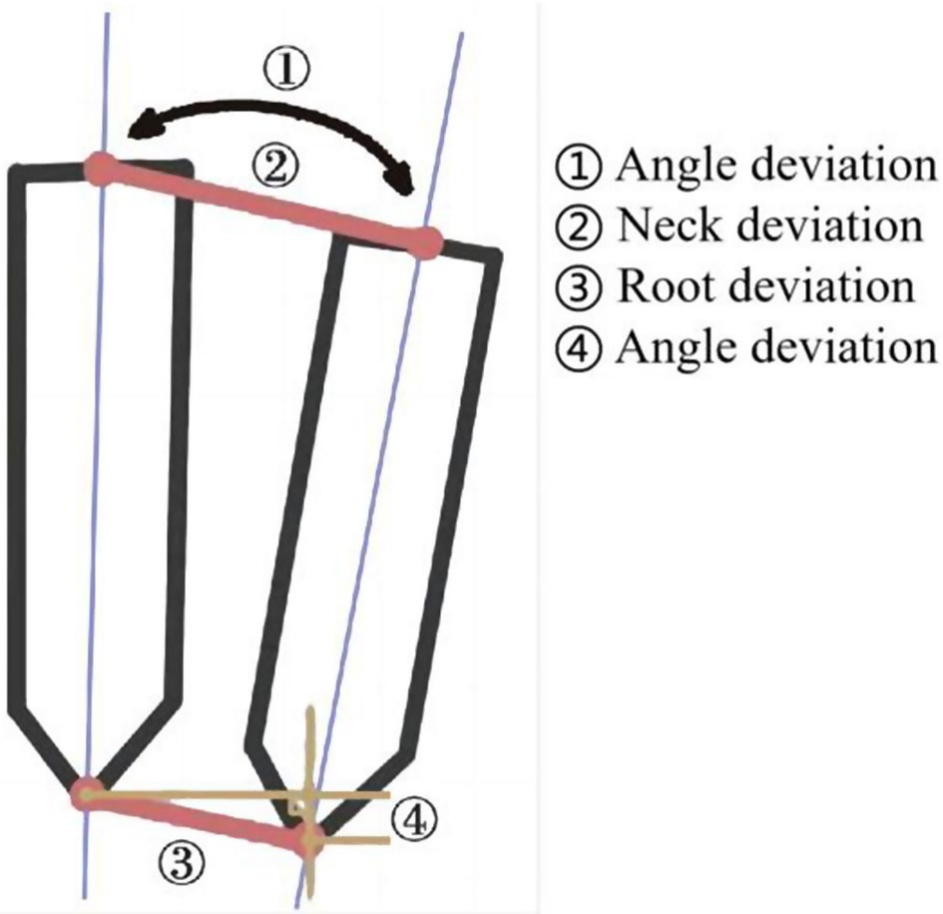
Implant deviation measurement model diagram

1. Initial Stability: To assess the initial stability of the implants, the implant torque was utilized, and any occurrence of inferior alveolar nerve injury was meticulously recorded.
2. Distance to Inferior Alveolar Nerve Canal: Immediately post-surgery, Cone-Beam CT (CBCT) scans were conducted to measure the distance between the implant root end and the inferior alveolar nerve canal.
3. Depth of Implant Placement: CBCT scans, performed immediately after surgery, were employed to measure the distance between the implant root end and the alveolar crest. Notably, all implants in this study were either bone-level or endosteal.
4. Implant Deviation: Post-surgery CBCT scans were imported into accuracy verification software (EPED Group, Taiwan). A 3D reconstruction model was generated and matched with the preoperative model. The deviation between the actual implant position and the preoperative design position was measured at the neck, root, depth, and angle, using postoperative CT data and the preoperative planning scheme, as illustrated in Fig. 6. This comprehensive analysis provided insights into the accuracy of the implant placement in terms of its spatial relationships.

### Statistical assessment

Statistical analyses were conducted using IBM SPSS version 22.0 for Windows (IBM Corp., Armonk, NY, USA). The measured data, all adhering to a normal distribution, were presented as mean ± SD. Analysis involved 1- or 2-way ANOVA and Student’s t-test. A *P*-value was < 0.05 was considered to indicate statistical significance.

## Results

### Initial stability

All implantations were successfully completed with favorable initial stability. The insertion torque for all implants exceeded 15 N.cm, with an average torque of 22.53 ± 5.93 N.cm. Specifically, the implant torque in the navigation and guide plate groups exceeded 25 N.cm. Notably, no instances of lower lip numbness were reported among the patients. Postoperative Cone-Beam CT (CBCT) scans revealed that all implants successfully avoided the inferior alveolar nerve canal, confirming the precision and safety of the implant placement procedures in the posterior mandibular region.

### Distance between implant and inferior alveolar nerve canal

**Fig. 7:**
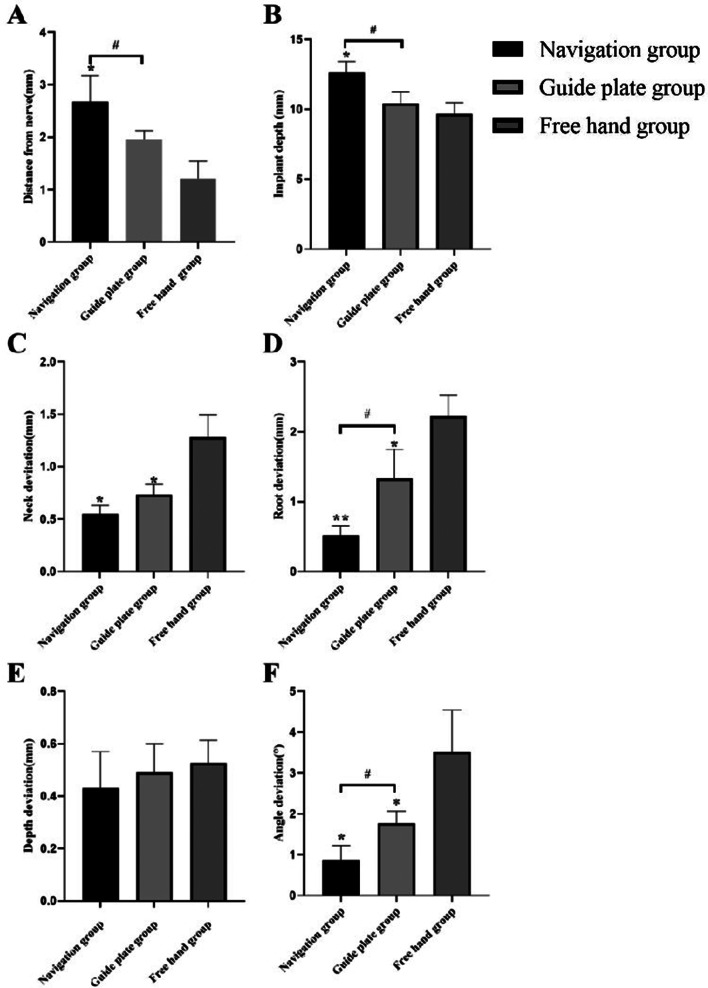
Measurement of implant deviation, distance from nerve and implant depth. **A** Distance between implant and inferior alveolar nerve canal; **B** Depth of implant placement; **C** Neck deviation; **D** Root deviation; **E** Depth deviation; **F** Angle deviation. * : *P* < 0.05, compared with free hand group; # : *P* < 0.05, navigation group compared with guide plate group

The results for the dynamic navigation group, static guide plate group, and freehand implant group were 2.68 ± 0.50 mm, 1.97 ± 0.17 mm, and 1.21 ± 0.33 mm, respectively (Table 1). The distance between the implant and the inferior alveolar nerve in the navigation group was significantly longer than that in the guide plate group and freehand implant group (*P* < 0.05). Additionally, the template group showed a greater distance compared to the freehand implant group, but the difference was not statistically significant (*P* > 0.05) (Fig. 7A). This analysis provides quantitative insights into the spatial relationships between the implants and the inferior alveolar nerve, highlighting the distinct outcomes associated with each implantation technique.

### Depth of implant implacement

The results for the dynamic navigation, static guide plate, and freehand implant groups were 12.73 ± 0.93 mm, 11.26 ± 0.69 mm, and 10.26 ± 1.41 mm, respectively (Table 1). The implant depth in the navigation group was significantly greater than that in the guide plate and freehand implant groups (*P* < 0.05). Moreover, the implant depth in the guide plate group was larger than that in the freehand implant group, though this difference was not statistically significant (*P* > 0.05) (Fig. 7B). These findings elucidate the varying implant depths associated with different placement techniques, emphasizing the significance of dynamic navigation in achieving greater depth in the posterior mandibular region.

### Implant deviation

#### Neck deviation

The neck deviations in the navigation, guide plate, and freehand implant groups were 0.56 ± 0.07 mm, 0.73 ± 0.10 mm, and 1.26 ± 0.13 mm, respectively (Table 1). The neck deviation in both the navigation and guide plate groups was significantly less than that in the freehand implant group, demonstrating a statistically significant difference (*P* < 0.05). However, there was no significant difference in the neck deviation between the navigation group and the guide plate group (*P* > 0.05) (Fig. 7C). This analysis highlights the superior precision in neck deviation associated with navigation and guide plate techniques compared to freehand implantation in the posterior mandibular region.

#### Root deviation

The root deviations in the navigation, guide plate, and freehand implant groups were 0.52 ± 0.13 mm, 1.33 ± 0.42 mm, and 2.22 ± 0.30 mm, respectively (Table 1). The root deviation in the navigation group was significantly smaller than that in both the guide plate and freehand implant groups, demonstrating a statistically significant difference (*P* < 0.05). Furthermore, the root deviation in the guide plate group was significantly less than that in the freehand implant group, with a statistically significant difference (*P* < 0.05) (Fig. 7D). These results underscore the enhanced precision in root deviation achieved through navigation and guide plate techniques compared to freehand implantation in the posterior mandibular region.

#### Depth deviation

The depth deviations in the navigation, guide plate, and freehand implant groups were 0.43 ± 0.14 mm, 0.49 ± 0.11 mm, and 0.53 ± 0.09 mm, respectively (Table 1). Importantly, no significant difference in the depth deviation was observed among the navigation group, the guide plate group, and the freehand implant group (*P* > 0.05) (Fig. 7E). This indicates comparable precision in terms of depth deviations across the three implantation techniques employed in the posterior mandibular region.

#### Angle deviation

The angle deviations in the navigation, guide plate, and freehand implant groups were 0.88 ± 0.45°, 1.77 ± 0.30°, and 3.52 ± 1.03°, respectively (Table 1). Significantly smaller angle deviations were observed in the navigation group compared to both the guide plate and freehand implant groups, indicating a statistically significant difference (*P* < 0.05). Additionally, the angle deviation in the guide plate group was significantly smaller than that in the freehand implant group, with a statistically significant difference (*P* < 0.05) (Fig. 7F). These findings emphasize the superior precision in angle deviations achieved through navigation and guide plate techniques in contrast to freehand implantation in the posterior mandibular region.

## Discussion

The rapid advancement of medical imaging equipment, coupled with the swift progress of 3D printing technology and surgical simulation planning software, has propelled dental implantology towards heightened safety and precision. The digital-assisted implant system involves preoperative design of the three-dimensional implant position using implant planning software in conjunction with imaging data. Subsequently, this design plan is utilized for the precise implementation of the implant, this is facilitated through either a digital guide plate or a dynamic navigation system. This approach signifies a transformative shift towards digitally guided and technologically enhanced practices in dental implant surgery, ensuring accuracy and efficiency in the placement process [15, 16]. In comparison to conventional free-hand implantation methods, the utilization of digital guide plates and dynamic real-time navigation stands out for significantly enhancing the accuracy and safety of oral implant procedures. These advanced technologies provide precise guidance throughout the implantation process, minimizing deviations and improving overall outcomes in terms of implant placement accuracy and patient safety [17, 18]. Nevertheless, there is a notable absence of studies specifically focusing on navigation-assisted immediate implantation following the extraction of mandibular posterior teeth. The inclusion of dynamic real-time navigation in this study holds practical clinical significance, particularly in the context of immediate implantation post-tooth extraction in the mandibular posterior region. This research addresses a critical gap in existing literature, contributing valuable insights that can inform and enhance clinical practices in this specific area of dental implantology.

In this study, the implementation of dynamic real-time navigation for immediate implantation following the extraction of mandibular posterior teeth not only proves to be a time-saving approach but also eliminates the need for guide plate production. This highlights a streamlined and efficient process, offering a potentially more straightforward and cost-effective alternative in comparison to traditional guided implantation methods [10, 12]. Moreover, this approach addresses the issue of water cooling associated with guide plates, providing a solution that is particularly well-suited for immediate implant placement (IIP) in the posterior region under diverse clinical conditions [9]. The utilization of dynamic real-time navigation offers a versatile and effective strategy to enhance the precision and efficiency of implant procedures, mitigating challenges associated with traditional guide plates [19]. During the implantation process, dynamic real-time navigation enables the visualization and real-time adjustment of implant design. This includes the ability to modify the implantation point, angle, depth, and overall implantation path. This real-time adaptability enhances the surgeon’s control and precision, allowing for on-the-spot adjustments based on specific anatomical considerations and ensuring optimal implant placement in the mandibular posterior region [20]. Therefore, for surgeons, dynamic navigation demonstrates favorable application characteristics and stands as an advanced assisted implantation method. Its real-time visualization, adjustability, and capacity to address various challenges make it a valuable tool in the hands of clinicians, enhancing precision and efficiency in the implantation process, particularly in the context of immediate placement after mandibular posterior tooth extraction.

Ensuring initial stability is a paramount consideration in immediate implant placement (IIP) [21, 22]. The findings of this study affirm that all implants achieved commendable initial stability, with insertion torques exceeding 15 N.cm. Notably, the implant torque for both the navigation and guide plate groups surpassed 25 N.cm. This aligns with clinical studies where dynamic navigation and static navigation plates were employed to guide oral implants, consistently yielding implant torsion values exceeding 25 N.cm. These results underscore the effectiveness of dynamic navigation in achieving robust initial stability, a critical factor in the success of immediate implantation procedures [23]. The observed outcome aligns consistently with the results of the current study. Consequently, it can be inferred that both dynamic navigation and static templates are capable of achieving commendable primary stability in the immediate implant placement (IIP) of mandibular posterior teeth. The findings suggest that navigation-assisted IIP in the mandibular posterior region surpasses free-hand implantation, demonstrating superior primary stability. This reinforces the notion that dynamic navigation, with its real-time guidance and adjustments, contributes to enhanced stability during the critical early stages of implantation.

Avoiding injury to the inferior alveolar nerve is a crucial concern in immediate implant placement within the posterior mandibular region. In this study, measurements of the distance from the root end of the implant to the inferior alveolar nerve revealed distances of 2.68 ± 0.50 mm, 1.96 ± 0.17 mm, and 1.21 ± 0.33 mm for the navigation group, guide plate group, and free-hand group, respectively. Significantly, the distance in the navigation group exceeded that in the guide plate and free-hand implant groups, with a statistically significant difference. These findings indicate that navigation-assisted implant placement effectively ensures a safe distance between the implant root and the inferior alveolar nerve, optimizing the use of remaining bone for robust primary stability while avoiding nerve damage.

Dynamic navigation proves advantageous in preventing damage to critical anatomical structures such as the inferior alveolar nerve due to two key factors. Firstly, the preoperative design software in the navigation system utilizes comprehensive patient imaging data for precise implant design, minimizing the risk of encountering the inferior alveolar nerve. Secondly, the dynamic navigation system’s visualization capabilities allow the surgeon to observe and real-time adjust the three-dimensional positional relationship between the implant and the inferior alveolar nerve during the implantation process [8, 10]. This real-time adaptability contributes to heightened precision, ensuring a safer and more controlled implant placement in proximity to sensitive anatomical structures.

This study delves into the investigation of implant placement depth, recognizing that deeper implant placement optimizes alveolar bone utilization [24]. The findings revealed depths of implant placement in the navigation, guide plate, and free-hand implant groups as 12.73 ± 0.93 mm, 11.26 ± 0.69 mm, and 10.26 ± 1.41 mm, respectively. Notably, the depth in the navigation group exceeded that in both the guide plate and free-hand implant groups, with a statistically significant difference. The utilization of navigation technology proves advantageous in maximizing residual bone volume within the alveolar bone, enabling the implantation of longer implants. This approach, particularly beneficial in cases of limited local alveolar bone post-tooth extraction, facilitates deeper implantation to secure a minimum of 3 mm of bone around the implant root, meeting the prerequisites for achieving robust initial stability [25]. The technical challenges associated with traditional free-hand implantation in the posterior mandibular region, compounded by the presence of the inferior alveolar nerve and constraints on the surgical field, make it challenging to achieve deep implant placement. Furthermore, the limited mouth opening in the posterior region adds to the difficulty. In comparison, the navigation group demonstrated an advantage, overcoming these challenges by facilitating deeper implant placement and utilizing a greater amount of alveolar bone. This underscores the efficacy of navigation-assisted implantation in addressing anatomical complexities and limitations associated with traditional free-hand methods, particularly in the posterior mandibular region [26].

Digital technology plays a crucial role in aiding implant placement, and achieving high accuracy is a pivotal concern in its application. The study results demonstrated that the neck, root, and angle deviations in the navigation group were significantly smaller than those in the free-hand implant group. Moreover, both root and angle deviations in the navigation group were significantly smaller than those in the guide plate group, with statistically significant differences. These findings highlight the substantial accuracy advantage of navigation-assisted immediate implant placement in the posterior mandibular region. The precision afforded by digital navigation technology contributes to improved outcomes and reinforces its efficacy as a valuable tool in enhancing the accuracy of implant placement procedures.

Additionally, several studies have conducted comparisons between dynamic navigation assistance and free-hand implantation. While these studies may not specifically focus on immediate implantation, they provide insights into the accuracy disparities between dynamic navigation assistance and free-hand implantation [27]. One such randomized controlled clinical trial specifically compared the accuracy of dynamic navigation and free-hand-assisted oral implantation in the posterior maxilla. These comparisons contribute to the broader understanding of the precision advantages offered by dynamic navigation in implant procedures, emphasizing its potential benefits over traditional free-hand methods [28]. The findings from this study align with existing research, as evidenced by another clinical trial comparing dynamic navigation-assisted implantation to free-hand implantation. In both studies, the neck, root, and angle deviations in navigation-assisted implantation were significantly lower than those in free-hand implantation. Furthermore, an additional clinical trial investigated linear deviations at the implant neck and root, along with angular deviations [29]. The outcomes revealed that the neck and angle deviations in the navigation group were significantly smaller than those in the free implant group, with no differences found in apex vertical deviation. Collectively, these consistent results across studies underscore the superior accuracy of navigation-assisted implantation when compared to free-hand implantation.

Furthermore, other studies have undertaken a comparative analysis of implant deviations among dynamic navigation-assisted implantation, static guide plate-assisted implantation, and free-hand implantation [12, 13]. Consistently, these studies reported that the neck, root, and angle deviations in dynamic navigation-assisted and static guide plate-assisted implantation were smaller than those in free-hand implantation, corroborating the findings of the present study. However, these studies did not identify significant differences in the accuracy between navigation-assisted and template-assisted implantation [30, 31]. This may be attributed to the fact that these studies did not specifically focus on mandibular posterior dental implants and immediate implant placement (IIP). Given the complex conditions of IIP extraction sockets and the challenges associated with guide plate placement, particularly in affecting the surgical field, dynamic navigation-assisted implantation, with its real-time and visualization advantages, exhibited superior accuracy in immediate implant placement in the posterior mandibular region compared to static guides.

Combining the findings from the aforementioned studies and the current investigation, a conclusive observation can be drawn. The accuracy of dynamic navigation-assisted implantation in the posterior mandibular region, regardless of whether it involves immediate or non-immediate implantation, surpasses that of static guide plate-assisted and free-hand implantation procedures. This consistent pattern across various studies underscores the reliability and precision offered by dynamic navigation technology in optimizing implant placement outcomes in the challenging anatomical context of the posterior mandibular region.

Several limitations to this study should be noted. The study is based on a retrospective chart review of medical records, which may contain errors or inconsistencies in the documentation of variables. Although we took steps to ensure the accuracy of the data by reviewing the data collection forms and conducting double data entry, there may still be some errors or missing data. Prospective randomized controlled trials are needed to further verify the clinical effect of dynamic navigation assisted dental implantation.

## Conclusions

Dynamic navigation for IIP in the posterior mandible provides the benefits of vision and accuracy, which can successfully prevent inferior alveolar nerve damage. It may take full use of the remaining bone mass to provide good primary stability.

## Data Availability

The data that support the findings of this study are available from the corresponding author upon reasonable request.

## Abbreviations

IIP: immediate implant placement
CBCT: Cone beam computed tomography
STL: Standard Tessellation Language
